# Quantification of Soluble or Insoluble Fractions of *Leishmania* Parasite Proteins in Microvolume Applications: A Simplification to Standard Lowry Assay

**DOI:** 10.1155/2020/6129132

**Published:** 2020-03-13

**Authors:** Bhagya Deepachandi, Sudath Weerasinghe, Thisira Priyantha Andrahennadi, Nadira D. Karunaweera, Nadeeja Wickramarachchi, Preethi Soysa, Yamuna Siriwardana

**Affiliations:** ^1^Department of Parasitology, Faculty of Medicine, University of Colombo, Colombo 00800, Sri Lanka; ^2^Department of Biochemistry and Molecular Biology, Faculty of Medicine, University of Colombo, Colombo 00800, Sri Lanka; ^3^National Science Foundation, 47/5 Maitland Place, Colombo 00700, Sri Lanka

## Abstract

Protein quantification is often an essential step in any research field that involves proteins. Although the standard Lowry assay and its modifications are most abundantly used in protein quantification, the existing methods are rigid or often demonstrate nonlinearity between protein concentration and color intensity. A method for fast and accurate qualitative and/or quantitative determination of total soluble/insoluble proteins or micro-well plate immobilized proteins isolated from *Leishmania* parasites in microvolumes was described in the current study. Improvements in cost-effective techniques are necessary to increase the research outputs in resource-limited settings. This method is a modification to the established Lowry assay for protein quantification. Concentrations of unknown samples were calculated using a standard curve prepared using a standard series of bovine serum albumin (BSA). The optimized reagents were 2 N NaOH (sodium hydroxide), 2% Na_2_CO_3_ (sodium carbonate), 1% CuSO_4_ (copper sulfate), 2% KNaC_4_H_4_O_6_ (potassium sodium tartrate), and 2 N Folin and Ciocalteu's phenol. This modified protein assay was sensitive for quantifying *Leishmania* proteins in a total crude extract or in a soluble fraction within the approximate range of 10–500 *μ*g/ml (1–50 *μ*g/assay) and showed a linearity between color intensity and concentration of the protein. This is an easier, fast, and accurate method for quantifying proteins with microvolumes in a cost-effective manner for routine use in research laboratories in resource-limited settings.

## 1. Introduction

Leishmaniasis is a vector-borne parasitic disease with wider geographical distribution in the world. The disease is caused by the parasitic protozoa of the genus *Leishmania* [1]. The disease management is challenging due to nonpathognomonic symptoms and significant toxicity of treatments. Hence better patient management is required with wider cases distribution. In such situations, majority of research scientists are working on developing tools or biomarkers based on *Leishmania* protein antigens for diseases diagnosis, prognosis, or therapeutic applications where parasitic antigen preparations are involved and protein quantification is necessary [2–4].

Protein quantification is also required in other different clinical or research applications. Therefore, many researchers and commercial institutions have established different protein assays for protein quantification. The method appropriateness depends on procedure time, requiring quantity of a protein sample, accuracy, reproducibility, and cost.

Among most common protein assays reported to date, Lowry protein assay [5] and Biuret assay [6] are the two established and oldest methods widely used for protein quantification. In 1972, Lowry assay has been modified to yield a higher color with a linear relationship between concentration of sample and color intensity [7]. After 1972, the standard Lowry protein assay has been modified several times by different research groups. They increased accuracy of protein quantification in presence of interfering chemicals, enhanced the protocol for rapid quantitative recovery of soluble and membrane proteins from interfering substances, adapted for use with 96-well micro-titer plates and an automatic microplate spectrophotometer, and enhanced optical density that reaches a maximum and remains constant for a sufficient period [8–11].

Also in 1976, Bradford et al. have described a new method for protein quantification by providing a reagent which comprises the dye Coomassie Brilliant Blue. But it is adversely affected by the presence of detergents in sample or wide protein-to-protein variation [12, 13]. There are several protein assays described by different research groups subsequently. Those included an assay using a reagent of Coomassie Brilliant Blue G250 dye in perchloric or hydrochloric acid, an assay using the reaction of protein with alkaline copper with bicinchoninic acid (BCA), an assay which can be used with a multilayer dry analytical element, a modified assay of BCA protocol with utilizing a microwave oven to irradiate samples and a process for total solid phase- or microparticle-immobilized proteins [14–18].

Recently more advanced peptide and/or protein quantification methods were developed for the use in mass spectrometry and for electrochemical quantification of proteins in medical applications [19, 20]. Also there are several trademarks for protein assays which were developed by well-established commercial suppliers [21–23].

In the current study, we describe a cost-effective and highly accurate modification to standard Lowry assay for quantifying both total soluble and crude protein extracted from *Leishmania* parasites with a minimal assay time. The assay is useful in resource-limited settings.

## 2. Materials and Methods

### 2.1. Instrumentation, Materials, and Reagents

Absorbance measurements were obtained by Shimadzu UV 1601 UV/visible spectrophotometer (Shimadzu Corporation, Kyoto, Japan), Thermo electron corporation Multiskan EX microplate reader, and Epoch 2 microplate spectrophotometer (BioTek instruments). Micropipettes (0–20 *μ*l, 20–200 *μ*l, and 100–1000 *μ*l Nichipet EXII micropipettes from Nichiryo), micro-well plates (96 wells) (Sterilin, Tentorio, Italy), and the reagents required for cell culturing [penicillin-streptomycin (Penstrep), heat inactivated fetal bovine serum (HI-FBS), medium 199 Hank's balanced salts (M199)] were used (Gibco Life Technologies, Grand Island, USA). All other chemicals and reagents, including sodium phosphate dibasic (Na_2_HPO_4_), sodium phosphate monobasic (NaH_2_PO_4_), sodium chloride (NaCl), potassium chloride (KCl), potassium phosphate monobasic (KH_2_PO_4_), sodium carbonate (Na_2_CO_3_), copper sulfate (CuSO_4_), potassium sodium tartrate (KNaC_4_H_4_O_6_), sodium hydroxide (NaOH), Folin and Ciocalteu's phenol reagent, bovine serum albumin (BSA/fraction V), were from Sigma-Aldrich (Now known as Merck, Saint Louis, Missouri, USA).

### 2.2. Preparation of Standards

BSA was used as the reference standard. The BSA standard samples were prepared with the same reagent used for the unknown samples [e.g., deionized water, 1XPBS (1X phosphate-buffered saline), lysis buffer with detergent (e.g., 1% triton X-100), or lysis buffer without detergent]. In this study, concentration of BSA stock solution was 1 mg/ml which was achieved by dissolving 1 mg of BSA in total of 1 ml of deionized water.

### 2.3. Protocol for Lowry Assay Carried Out in a Micro-Well Plate

A dilution series of BSA (10 to 500 *μ*g/ml of BSA) and unknown sample (100 *μ*l) were added to separate wells and mixed with 20 *μ*l of NaOH (2 N) in a plate shaker for 10 minutes. A volume of 100 *μ*l of reagent mixture A (2% Na_2_CO_3_, 1% CuSO_4_, and 2% KNaC_4_H_4_O_6_ in 100 : 1 : 1 ratio) was added to each well and mixed well for 5 minutes followed by incubation at room temperature for 10 minutes. Folin and Ciocalteu's phenol reagent (2 N, 20 *μ*l) was added, mixed well immediately, and incubated at room temperature in dark conditions for 30 minutes. Absorbance was read at 650 nm using a microplate reader.

### 2.4. Method Validation and Data Analysis

Method validation was carried out according to guidelines for bioanalytical method validation distributed by Food and Drug Administration (FDA), USA [24]. Selectivity of the assay was assessed by evaluating matrix effects. Accordingly, parallelism of diluted BSA standards was evaluated and standard curve was analyzed. Nonspecific binding was determined using a blank matrix (without analyte). Absorbance value measured for the blank matrix was reduced from absorbance values measured for each matrix with analyte (BSA standards), thereby avoiding any interference coming from the matrix and increasing the selectivity of the assay. Repeatability of the assay was determined using ten determinations for each concentration and thereby determined the accuracy of the assay. Six different concentrations of BSA standards (10, 30, 100, 150, 300, and 500 *μ*g/ml) were performed and M (mean), SD (standard deviation), M + 2SD (upper limit), M-2SD (lower limit), and CV (coefficient of variation) were calculated. Intrabatch (within run) and interbatch (between runs) precision or repeatability were further determined using ten determinations at six different concentrations of BSA as described above, by running at the same day in the same plate and by performing at 20 different days, respectively. If absorbance value for any concentration of BSA at any occasion was observed outside of the accepted limits (between M + 2SD and M-2SD), the values were rejected and assay was repeated. Interbatch precision was also measured with regard to different time (20 different days), different equipment (using Thermo electron corporation Multiskan EX microplate reader and Epoch 2 microplate spectrophotometer from BioTek instruments), and different reagents (five different batches of stock reagents prepared) and in two different laboratories. Lower limit of quantification (LLOQ) was established using the six selected lowest concentrations of BSA (5, 10, 30, 60, 80, 100 *μ*g/ml) with ten determinations for each concentration. Upper limit of quantification (ULOQ) was defined using the highest standard with reproducible, high precision, and high accuracy. Linearity of the assay was determined using the standard curve created with six different concentrations of BSA including LLOQ, low, medium, and high concentrations in duplicate in each run. SD_0_ (intercept of the standard curve at zero concentration) was used for evaluating limit of detection (LOD) and limit of quantification (LOQ) of the assay. The values of 3 × SD_0_ and 10 × SD_0_ were calculated as LOD and LOQ, respectively [25]. Chemical stability of stock solutions and the standard were further determined to assess stability of new assay [24]. The validated assay was further analyzed for a BSA protein sample with unknown concentration and compared with standard Lowry assay described below which was carried out in large scale and already established within the home laboratory [5, 7].

### 2.5. Protocol for Standard Lowry Assay Carried Out in Micro-Centrifuge Tubes

A dilution series of BSA (10 to 500 *μ*g/ml of BSA) and unknown sample (100 *μ*l) were added to micro-centrifuge tubes separately and mixed well with 100 *μ*l of NaOH (2 N). The mixture was incubated at 100°C for 10 minutes followed by cooling to room temperature. A volume of 1 ml of reagent mixture A (2% Na_2_CO_3_, 1% CuSO_4_, and 2% KNaC_4_H_4_O_6_ in 100 : 1 : 1 ratio) was added to each tube and mixed well. The tubes were incubated for 10 minutes at room temperature. Folin and Ciocalteu's phenol reagent (2 N, 100 *μ*l) was added, mixed well immediately, and incubated at room temperature in dark conditions for 30 minutes. The final volume of reacting mixture was 1300 *μ*l in each tube. Absorbance was read at 750 nm using a UV spectrophotometer.

### 2.6. Quantification of Leishmania Parasite Proteins Using the New Assay

*Leishmania* promastigotes were grown in complete M199 media supplemented with 10% HI-FBS and 0.1% Penstrep [26]. Parasites at late log phase with an average density of about 1 × 10^7^ cells/ml were harvested and pellets were stored at −20°C until use. Crude *Leishmania lysate* was extracted from the harvested promastigotes of *Leishmania* using freeze-thawing method [27]. The pellet was washed four times in cold 0.01 M PBS, pH 7.4, and resuspended at a concentration of 1.0 g of cell pellet in 2.0 ml of cold 0.01 M PBS, pH 7.4. Subsequently, the suspension was freeze-thawed (freezing for 30 seconds in liquid nitrogen and thawing at room temperature) for three times. The suspension contained the total crude lysate and it was further centrifuged at 10,000 g for 10 minutes and supernatant containing soluble fraction of crude lysate was separated. Protein contents of extracted crude lysate and soluble fraction of *Leishmania* crude lysate were estimated using the validated micro-Lowry assay (we used deionized water for preparing BSA standards since only 2–5 *μ*l of unknown sample/crude antigen was enough for quantification and it was prepared to 100 *μ*l using deionized water).

### 2.7. Monitoring the Efficiency of Different Buffers for Antigen Coating to Micro-Well Plates Using the New Assay

Three different antigen coating buffers in enzyme-linked immunosorbent assay (ELISA) were analyzed for selecting the best coating buffer for subsequent applications of *Leishmania* antigen using ELISA. The binding of a protein to the polystyrene surface of micro-well plate is usually done by hydrophobic interactions which happens in basic, neutral, and acidic buffers. PBS (1X, pH 7.4), phosphate buffer (0.02 M, pH 7.8), and carbonate buffer (0.05 M, pH 9.6) were used as coating buffers for the study since those were widely used by other researchers working on *Leishmania* [28–30]. *Leishmania* antigen preparation and quantification were done as described above. Equal amount of antigen (3 *μ*g/well) was used for coating the wells. Ten replicates were carried out for each coating buffer. The antigen was added to each well (3 *μ*g/100 *μ*l/well) and incubated overnight at +4 C refrigerator. Following overnight incubation, the wells were washed three times with PBS (1X, pH 7.4) to remove unbound materials and plate was used for protein quantification assay. The described protein assay was carried out for antigen coated wells. A standard series of BSA dilutions were carried out within the same plate parallel to the coated wells as described above. M, SD, and CV were calculated and analyzed for ten replicates carried out with three coating buffers and the best coating buffer with highest performance was selected for subsequent ELISA applications of *Leishmania* antigen.

## 3. Results

**Figure 1:**
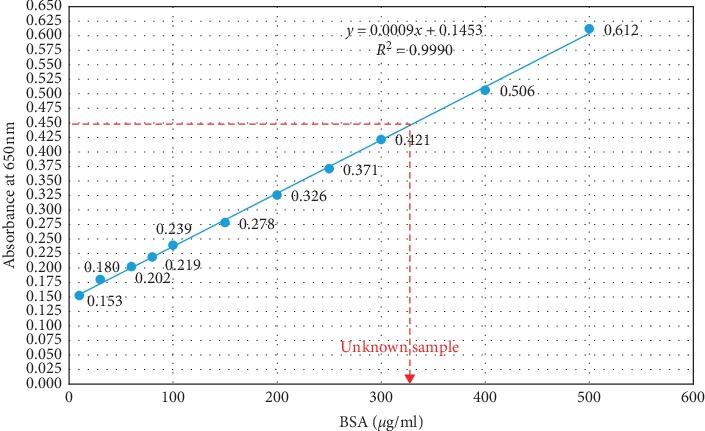
Standard curve constructed, at 650 nm, for a dilution series of BSA standard. The BSA concentration and absorbance value showed a linear relationship where *R*^2^ was 0.999.

The new assay showed a high selectivity for the analyte measured. According to the standard curve constructed using the results obtained for the dilution series of BSA (10 to 500 *μ*g/ml), the two variables of the assay, the BSA concentration and absorbance value at 650 nm, showed a linear relationship where the squared correlation coefficient, R^2^, was 0.999 (Figure 1).

The absorbance values for selected concentrations of BSA (10, 30, 100, 150, 300, 500 *μ*g/ml) at any given day were within the accepted limits (between lower and upper limits as shown in Table 1). The described simplified Lowry assay was 100% repeatable, having less than 10% CV, and therefore the assay showed a high accuracy for quantifying proteins.

**Figure 2:**
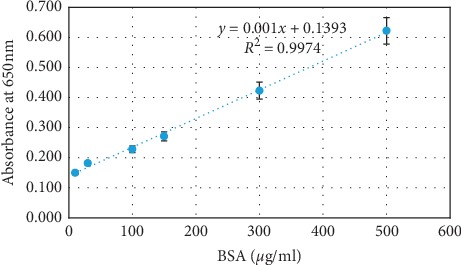
A standard curve constructed for six different concentrations of BSA standards. Vertical bars at each data point indicate the positive and negative error bars of each data point calculated according to SD of absorbance value at particular concentration of BSA.

Analysis of *R*^2^ of each standard curve constructed with intrabatch and interbatch repeatability assays showed *R*^2^ > 0.99 with <10% of CV. LLOQ and ULOQ were determined as 10 and 500 *μ*g/ml, respectively. SD_0_ was about 0.140 (0 *μ*g/ml) as shown in Figure 2. Therefore the LOD (=3 × SD_0_) and LOQ (=10 × SD_0_) were calculated as 0.420 (280 *μ*g/ml) and 1.400 (1260 *μ*g/ml), respectively.

According to manufacturer recommendations for the micro-well plate reader (Thermo electron corporation Multiskan EX), accurate range was typical value ± 1% (0–2.0 Abs) at 405 nm. Chemicals prepared for the assay including 2 N NaOH, 1% CuSO_4_, 2% KNaC_4_H_4_O_6_, 2% Na_2_CO_3_, and 2 N Folin were stable for more than three years when they were kept at room temperature and stored separately without mixing [31]. 2 N Folin should be kept under dark conditions avoiding direct sun light. BSA stocks were prepared at 1 *μ*g/ml and aliquoted and stored at −20°C avoiding repeat freeze-thaw cycles. Although aliquoted BSA can be used for more than one month, the preparation of fresh BSA before each experiment was recommended to increase the accuracy of the test.

The described method showed an accurate quantification of a protein with a concentration of 10–500 *μ*g/ml (1–50 *μ*g/assay) within the linear range of the standard curve, hence showing a medium sensitivity compared to the established protein assays (Table 2).

**Figure 3:**
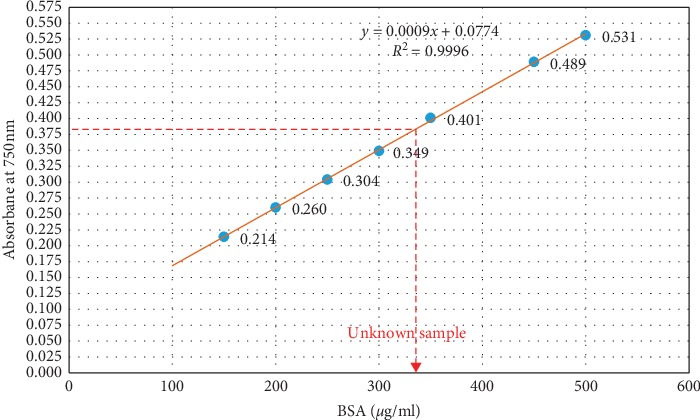
A standard curve constructed for seven different concentrations of BSA standards. The assay was performed according to the standard Lowry assay procedure carried out in large scale which was already established within the home laboratory.

**Figure 4:**
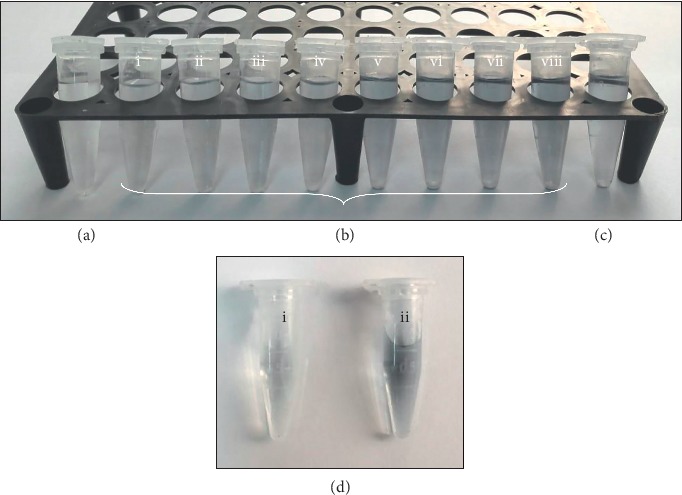
Standard Lowry assay carried out in micro-centrifuge tubes. (a) Reagent blank. (b) BSA standards with (i) 100 *μ*g/ml, (ii) 150 *μ*g/ml, (iii) 200 *μ*g/ml, (iv) 250 *μ*g/ml, (v) 300 *μ*g/ml, (vi) 350 *μ*g/ml, (vii) 450 *μ*g/ml, and (viii) 500 *μ*g/ml concentrations. (c) The BSA sample with an unknown concentration. (d) The samples treated with Lowry's reagent: (i) the blank and (ii) a crude protein extract of *Leishmania* parasites.

The concentration of unknown BSA sample showed absorbance values of 0.378 (at 750 nm) and 0.448 (at 650 nm) for standard Lowry assay (Figures 3 and 4) and newly optimized Lowry assay (Figure 1), respectively (the concentration of the unknown sample was shown in red color arrow in relevant figures). According to the respective standard curve of each assay, the concentration of BSA sample was calculated as 334 *μ*g/ml and 336 *μ*g/ml. Therefore, the assay performance of new assay was comparable to the standard method.

According to the new assay, yield of the total crude protein extract of *Leishmania* parasites was about 15 mg for 1 g of the parasite cell pellet. The new method was also tested for *Leishmania* crude antigens in 1X PBS (0.01 M PBS) and lysis buffer with triton X-100 (1%). The new method was effective for both and comparable results were observed.

Even though high amount of antigen was coated with phosphate buffer compared to PBS and carbonate buffer, the precision of 10 replicates was less with phosphate buffer compared with the other two buffers (Table 3). Variation of CV showed the precision/accuracy of each condition where phosphate buffer showed the highest CV and carbonate buffer showed the lowest CV of <10% which had a good precision compared to the other buffers. Therefore, carbonate buffer can be used for coating antigen where 1 *μ*g of antigen/well is enough for the subsequent ELISA applications.

## 4. Discussion

In spite of the availability of many established commercial kits for protein quantification at present, the efficiency of those kits is varied according to different applications. The cost of most products is very high. Therefore, it was vital to develop and validate a cost-effective protein quantification method for routine research purposes in resource-limited settings. As described above, assay validation was done using several standard parameters. Linearity is one of the most important characteristics for evaluation of the accuracy in assay validation. The developed test showed a high accuracy with *R*^2^ nearly equal to 1. Also the high reproducibility and repeatability, stability of reagents more than three years, and wider range of sensitivity (1–50 *μ*g/assay) further indicated appropriateness of the new method for the use in routine research.

When considering the reaction mechanism behind Lowry assay, Lowry et al. in 1951 described that protein reacts with alkaline copper an produces cuprous ions and reduces the Folin-Ciocalteu to produce a characteristic blue color when coupling a protein in buffered alkaline copper solution with Folin-Ciocalteu reagent containing phosphomolybdic and phosphotungstic acids [5]. Recently, Everette et al. suggested that the reaction mechanism involves both reduction of the Folin-Ciocalteu reagent and oxidation of aromatic residues, mainly tryptophan and tyrosine. Also experiments have shown that cysteine is also reactive to the Folin-Ciocalteu and cysteine residues in protein also contribute to the absorbance seen in the Lowry assay [32].

The standard Lowry method suffers from many disadvantages including nonlinearity of protein concentration and color intensity, rigidity of the method, poor reproducibility requiring precisely timed additions of reagent, immediate vortexing, and prolonged incubation [5]. In 1972, Hartree discovered a modified method for Lowry assay described in 1951 as to give a higher color yield with presence of direct proportionality between absorbances at 650 nm by incubating the protein sample in a more concentrated alkaline copper tartrate reagent at temperatures above ambient [7]. Although there were different methods developed as modifications to standard Lowry assay to carry out in micro-well plates for particular purposes of laboratories where large numbers of samples are processed and where a microplate spectrophotometer is already in use for other purposes, there were no methods developed so far to obtain linearity between protein concentration and color intensity with high accuracy [10, 23] (Table 4). The new method described here overcomes almost all of these problems without incubating at temperatures above ambient. In comparison to standard Lowry assay, the new method is carried out at small scale, using less volume of chemicals or reagents, BSA standards, and unknown samples. Also, in a micro-well plate, large number of samples can be quantified at the same time. This reduces the cost of chemicals or reagents, consumables, and time of experimental procedure in basic small scale laboratory setting. The new methodology avoids the requirement of purchasing commercial protein quantification kits. Cost per sample is approximately 2.8 USD by considering expenses for consumables, chemicals, and equipment.

## 5. Conclusions

This is a simplification to standard Lowy protein assay for quantifying parasite proteins (in crude extracts, soluble fractions, or immobilized to micro-titer plates) in a comparatively easy and accurate way. This is also a cost-effective method for utilizing in protein research works rather than using expensive kits or reagents.

## Figures and Tables

**Table 1:** Assessment of precision/accuracy of the assay.

	Absorbance values of different BSA (*μ*g/ml)					
Replicates	10	30	100	150	300	500
Mean (M)	0.150	0.182	0.228	0.271	0.423	0.622
Standard deviation (SD)	0.006	0.006	0.011	0.015	0.028	0.044
2SD	0.012	0.013	0.022	0.030	0.056	0.088
Upper limit (M + 2SD)	0.162	0.195	0.249	0.301	0.479	0.710
Lower limit (M − 2SD)	0.138	0.170	0.206	0.241	0.367	0.534
Coefficient of variation (CV)	3.970	3.495	4.828	5.451	6.613	7.077

**Table 2:** Sensitivity ranges of most established protein assay methods.

Protein assay method	Sensitivity range
Biuret method [6]	1–10 mg
Hartree's modified Lowry assay [7]	15–110 *μ*g
Bradford or Coomassie brilliant blue method [13]	1–200 *μ*g
Colloidal gold method [18]	20–640 ng
Bicinchoninic acid assay (Smith or BCA method) [15]	0.2–50 *μ*g
Lowry protein assay [11]	2–100 *μ*g
The proposed method	1–50 *μ*g

**Table 3:** Assessment of the precision of the antigen coating procedure using three different coating buffers.

Replicates	PBS (1X, pH 7.4)	Phosphate buffer (0.02 M, pH 7.8)	Carbonate buffer (0.05 M, pH 9.6)
1	0.022	0.030	0.023
2	0.027	0.081	0.018
3	0.028	0.028	0.023
4	0.026	0.029	0.022
5	0.021	0.020	0.023
6	0.023	0.030	0.019
7	0.022	0.057	0.018
8	0.023	0.046	0.021
9	0.030	0.021	0.021
10	0.026	0.020	0.019
M	0.025	0.036	0.021
SD	0.003	0.020	0.002
CV	12.018	54.379	9.940
Ag, *μ*g/ml	12.981	20.125	10.438
Mean amount of coated antigen (*μ*g)	1.298	2.013	1.044

**Table 4:** Comparison of reporting method with other commercially available Lowry assay kits.

Feature	Proposed method	Some commercially available Lowry assay kits (brand specifications are not provided)		
		Assay A	Assay B	Assay C
Linear quantification range	10–500 *μ*g/ml	Nonlinear	0–10 *μ*g/ml	5–100 *μ*g/ml
Use of micro-well plate methods	Yes	Yes	Yes	Yes
Total reaction volume per well	240 *μ*l	260 *μ*l	320 *μ*l	130 *μ*l
Total incubation time	55 min	41 min	45 min	40 min

## Data Availability

The data supporting the conclusions of this article are included within the article. Additional details are available from the corresponding author upon reasonable request.
